# Supplementary material for: Are carbon emissions trading and green financial instruments synergistic? -Comprehensive quantitative research based on content analysis
Source: PLoS One. 2024 Mar 7;19(3):e0298601. doi: 10.1371/journal.pone.0298601 (PMC10919601; doi:10.1371/journal.pone.0298601)
Supplement: S1 Data — (ZIP) [file pone.0298601.s001.zip › Data/地级市Z_F工作报告.zip!/┴╔─■/╠·┴δ╩╨/└┤╫╘╣1⁄2╓┌║┼í╛┬φ┐╦╩2╛▌═°í┐_2008.docx]

各位代表：

现在，我代表市人民政府向大会报告工作，请予审议，并请市政协委员和其他列席会议的同志提出意见。

一、2007年工作回顾

过去的一年，是铁岭发展史上又一个辉煌之年。我们在省委、省政府和市委的正确领导下，在市人大依法监督、市政协民主监督与大力支持下，团结和带领全市各族人民，坚持以邓小平理论和“三个代表”重要思想为指导，以科学发展观为统领，万众一心，坚韧不拔，只争朝夕，奋力拼搏，全面完成了市五届人大四次会议确定的各项任务目标，国民经济和社会事业在新起点上实现了新跨越。

——国民经济实现超常规跨越式发展，综合实力跨上新台阶。全市地区生产总值预计实现4O5亿元，增长2O．8％。其中，一二三产业增加值实现102亿元、185亿元和118亿元，分别增长10．2％、32％和12％。地区财政收入42．3亿元，增长28．8％。地方财政一般预算收入23亿元，增长41．3％。一般预算支出59．8亿元，增长32．7％。全社会固定资产投资完成304亿元，增长50％。城镇居民人均可支配收入8960元，增长20．5％。农民人均纯收入5000元，增长18．4％。金融存款余额366．2亿元，贷款余额309．8亿元，分别比年初增长9．4％和15．3%。

——沈铁工业走廊建设取得重大进展，传统农业地区正在向新型工业化城市迈进。重点工业园区呈现规划标准高、投入力度大、建设速度快的喜人局面，园区功能更加完善，环境更加优美，承载大项目的能力进一步增强。项目年活动取得历史性突破。共引进域外投资项目770个，实际到位资金237亿元，其中亿元以上项目93个。新批外商投资企业35家，实际利用外资1亿美元，增长2．1倍。可以说，2007年是铁岭历史上引进大企业集团和重大项目最多的一年。通过大唐集团能源化工项目的引进，不但优化了经济结构，增强了经济发展后劲，而且会加快我市走新型工业化道路的步伐。通过铁岭电厂二期、清河电厂“上大压小”，风力发电、开原雨润、赢德肉禽等一批项目的开工建设，新型能源基地和农产品精深加工基地建设获得了长足的进展。通过引进金刚水泥、天祥钢铁、原油储备等一批利税大、见效快的项目，做大了经济总量，提高了经济效益。通过数控机床、5O万伏输变电设备等一批高科技项目的引进，提高了沈铁工业走廊的科技含量，实现高新技术产品产值72亿元，增长50％。通过西丰铸造、凯尔重工等418个技改项目的实施，完成投资116亿元，争取省技改贴息和专项补助资金10231万元，增强了企业的自主创新能力，一批骨干企业迅速做大做强，销售收入超亿元的企业由上年的51户增加到7O户。通过实施节能减排工程，发展循环经济，万元生产总值综合能耗下降5．2％，主要污染物排放总量减少2％，空气达到国家二级质量标准343天，达标率94％。通过深化国有企业改革，玉深公司、铁岭钢厂等一批停产多年的企业资产盘活，重焕生机。通过大力培育产业集群，有色金属、先进制造、机械加工、橡塑制品、生物化工等产业集聚效应进一步显现。通过大力实施工业强市战略，工业化进程明显加快。规模以上工业增加值预计实现166亿元，增长35％；工业增加值占地区生产总值的比重达到43．2％，提高4．2个百分点。

——凡河新城区呈现崭新形象，城市建设步伐明显加快。经过各路建设大军昼夜兼程的紧张施工，5平方公里起步区基础设施建设全部完成，达到九通一平标准。15平方公里基础设施提前开工建设。各项水系全线贯通，凤冠山堆筑基本完成，莲花湖湿地恢复工程紧锣密鼓地进行。一次性开发1平方公里的“浅水湾一号”全面开工建设，凡河新村二期竣工并交付使用，技师学院、财税中心、铁岭县高中、九年一贯制学校、金融中心、市县市民服务中心等重点建设工程进展迅速，建筑工程开工面积177万平方米。广裕街改造、热源厂建设等1O项改造提升银州区功能的重点工程全部完成。特别是对城市环境进行了大规模的综合整治，城市面貌焕然一新。 “五城联创”取得可喜成果，我市被评为全国双拥模范城。副中心城市建设步伐明显加快，调兵山市被评为国家级园林城市，开原市晋升省级园林城市，西丰县县城改造成效显著。

——县域经济实力明显增强，新农村建设扎实推进。各县(市)区生产总值和财政一般预算收入平均增长30％以上。粮食生产再创历史新高，总产量达到69．3亿斤，保护地蔬菜、榛子、优质水稻、花生分别发展到65万亩、6O万亩、8O万亩和6O万亩。预计实现畜牧业产值116．5亿元，增长3 3％；肉蛋奶总产量93．2万吨，增长14％。农产品标准化生产面积达到320万亩，绿色和无公害农产品认证达到280个。新建固定资产投资2000万元以上农业产业化项目48个，实现农业产业化产值257亿元，增长21．8％。农村合作经济组织健康发展，西丰县永得利绿色蔬菜产业合作社在全省介绍了经验。成功承办国家和省科技下乡活动，引进农作物新品种100个，推广农业实用新技术2O项。完成“阳光工程”培训3万人，转移农村富余劳动力5．25万人。完成农建骨干工程264项，连续夺得省“大禹杯”。完成造林27．8万亩，开发榛林15万亩，铁岭榛子荣获“中华名果”称号。新建、改造农村公路1219公里，村通油路率达到85．1％。农机总动力178万千瓦，农业综合机械化水平达到48％。我市被省政府评为新农村建设优胜单位，开原市庆云堡镇被国家评为新农村建设示范区。

——服务业档次进一步提升，物流基地建设实现重大突破。由华南城集团投资5O亿元建设的铁岭东北物流城全面开工建设，一期工程年内即可竣工并投入运营。大商集团落户我市，西丰东北土特产品交易中心、开原五金城、建材城投入使用。新增“万村千乡市场工程”连锁店3 60个、新合作连锁店156个，初步形成了农村现代流通网络。商品市场繁荣活跃，兴隆大家庭等商贸流通企业购销两旺，社会消费品零售额实现130亿元，增长16．6％。外贸出口实现1．2亿美元，增长20％。成功组建了市商业银行和中小企业担保公司，建立了政策性融资、市场化融资和争取资金3个平台，金融对地方经济社会发展的支撑能力明显增强。旅游业日益兴旺，实现总收入3O．5亿元，增长52％。土地招拍挂更加规范，土地经营成效显著。房地产市场曰趋活跃，物业管理得到加强。粮食市场稳定有序。通信、邮政业快速发展。

——民生工程逐项落实，改革发展成果更多地惠及百姓。筹集资金3亿元，改造棚户区25万平方米，惠及棚户区居民2300户。筹集资金1．9亿元，培训下岗失业人员和进城务工农民35275人，安置实名制就业80058人，零就业家庭至少实现一人稳定就业。筹集社保资金10．57亿元，开通了“五险合一”，社保网络，确保了108603名企业离退休人员养老金按时足额发放。筹集资金1．3亿元，保证了87341户城乡低保对象的基本生活，新建、维修农村低保户危房1100户，及时为城市低保对象和大中专学生发放了副食补贴。筹集资金2382万元，提高了农村五保户的供养标准。筹集资金5386万元，落实105个扶贫开发项目，5．42万人实现脱贫。筹集资金2030万元，对11．8万受灾户及5．2万户城乡困难群众及时进行了救济。筹集资金5 3 0 0万元，提高了优抚对象的抚恤补助标准。筹集资金7 9 2 5万元，完成农村饮水安全工程426处，解决了2966万农村人口饮水安全问题。筹集资金335万元，进一步加强社区建设，银州区被省政府评为和谐文明社区示范区。筹集资金4500万元，支持西丰县完成了诚信水库主体工程。

——各项社会事业协调发展，人民生活质量进一步提高。教育年活动取得阶段性成果。新建农村九年一贯制学校11所，改造D级危房5．2万平方米。选派268名城镇教师到农村支教。职业教育办学水平得到提升，辽宁职业学院跻身省高职强校，西丰县职教中心迈入国家级重点行列。高等教育对外合作首开先河，铁岭师专派出了第一批赴美留学生。共收到社会各界捐资助教款1700多万元。通过开展教育年活动，全社会对教育的关切度、各级政府对教育的投入力度、校园面貌、师资队伍素质、学生身心素质发生深刻变化。积极探索政府购买公共卫生服务新模式，公共卫生服务体系更加健全，医疗服务监管工作得到加强。筹集资金1．27亿元，进一步改善农村医疗卫生条件，新建、改扩建乡镇卫生院71所，累计补偿参合农民患者227．27万人次，补偿资金9960万元。城市社区卫生服务中心发展到11个，服务站72个，我市被国家确定为社区卫生工作重点联系城市。文化事业繁荣活跃。秧歌戏《擂台情缘》荣获辽宁省首届政府文华奖，多幅工笔画和书法作品入选国展并获得奖励。成功举办了第二届城乡广场文化活动。投资600多万元，创建体育休闲广场125个，安装健身器材120套。成功举办了铁岭市第六届运动会。完成了200个广播电视村村通工程。继续稳定了低生育水平，人口素质进一步提升，出生人口性别比偏高问题得到有效遏制。社会主义荣辱观教育全面开展，群众性精神文明创建活动蓬勃兴起，公民思想道德建设提高到一个新水平。

——社会管理得到加强，社会总体保持和谐安定。深入排查调处社会矛盾纠纷，建立健全了信访工作长效机制和处置突发信访事件联动机。妥善处理了清河特钢“4·18”事故。安全隐患排查专项整治取得实效。切实加强市场监管，深入开展食品安全专项整治活动，采取有效措施稳定市场物价，有力地维护了市场经济秩序。应急管理体制和工作机制不断完善，应急保障能力得到加强。平安铁岭建设成绩显著，社会治安综合治理工作进一步加强。公安“三基”工作迈上新台阶，阳光警务工程取得良好社会效益，被评为全国十佳警务战略市。以行政审批制度改革、公共行政服务中心建设、政务公开等为重点的行政管理体制改革全面推进，行政效能得到提高。政府法制建设切实加强，依法行政全面推进，被省政府评为依法行政先进市。认真贯彻落实党的民族宗教政策，各民族团结和睦，宗教界稳定和谐。全年办理人大代表建议、政协提案4 04件，代表和委员满意率达到98％以上。

外事、侨务、台办、统计、审计、人事编制、综合执法、档案、工商、国税、地税、电业、规划、质量技术监督、出入境检验检疫、烟草、气象、人防、国家安全、司法行政、武警消防、新闻出版、地震、接待、科协、残联、商业保险、驻外联络、仲裁、市志、老龄等工作也都取得了新成绩。

各位代表！全市各项工作之所以能够实现新突破，开创新局面，关键就在于我们坚定不移地贯彻落实科学发展观，深入实施“五大战略”，全力推进“双轮驱动”，矢志不移，合力攻坚；关键就在于我们坚持一切从实际出发，求策于民众，问计于实践，更新发展理念，创新发展模式，大胆探索，不断完善；关键就在于我们团结一切可以团结的力量，调动一切可以调动的积极因素，激发广大干部群众的创业豪情，和衷共济，苦干实干。由于我们坚持以沈铁工业走廊建设为中心，全力推进工业化，一个“工业集中、产业集聚、土地集约、功能完善、管理科学、环境优美、生态良好”的新型工业化基地初具规模，一条生机勃勃的经济隆起带正在辽北大地迅速崛起。正因为沈铁工业走廊品牌越叫越响，铁岭传统农业地区的对外形象迅速改变。由于我们坚持以凡河新城区建设为重点，全力推进城镇化，一个崭新的现代化城市己具雏形，一座“中国北方水城”正在向世人展现独特魅力。正因为凡河新城区的拔地而起，“较大城市”才由一句幽默台词逐步变成现实。在此，我代表市人民政府，向辛勤工作在各条战线上的全市各族人民，向本届政府组成以来一直积极支持政府工作的市人大代表、市政协委员，向与政府肝胆相照、荣辱与共的各民主党派、工商联、无党派人士，向人民团体及社会各界人士，向解放军驻铁部队指战员和武警官兵、中省直驻铁单位，向来铁岭投资的广大客商，向所有关心支持铁岭建设与发展的海内外朋友表示衷心的感谢并致以崇高的敬意！

在看到成绩的同时，我们也清醒地认识到，全市经济社会发展还面临不少困难和问题，我们的工作与人民群众的期望相比还有一定差距。一是经济总量小、工业化水平低、城镇化水平低的局面没有得到根本改变，“ 一小两低”仍然是我市经济社会发展面临的主要矛盾。二是经济发展方式还比较粗放，经济发展质量还不够高，产业结构不尽合理，一产比重大，三产比重低，产业转型和优化升级仍然任重道远；节能减排和环境保护任务繁重。三是影响发展的体制机制障碍依然存在，国企改革遗留问题亟待解决，改革攻坚任务十分艰巨。四是人民群众收入水平还比较低，城镇居民人均可支配收入比全国平均水平低43OO多元，比全省平均水平低3OOO多元，城乡还有相当数量的贫困人口；经济社会发展不够协调，劳动就业、社会保障、社会分配、教育就学、看病难看病贵等事关群众切身利益的问题仍然较多。五是农村发展相对滞后，“三农”问题仍然比较突出。六是政府职能转变还没有完全到位，行政效能还有待提高，铺张浪费、吃拿卡要现象还一定程度存在。我们必须保持清醒的头脑，居安思危，高度重视这些困难和问题，并在改革和发展过程中认真加以解决。

二、2008年的主要工作

2008年，政府工作的指导思想是：全面贯彻党的十七大和中央经济工作会议精神，以邓小平理论和“三个代表”重要思想为指导，深入贯彻落实科学发展观，全面推进改革，扩大对外开放，提高自主创新能力，全力提升工业化、城镇化水平，做大经济总量，优化经济结构，提高经济效益，转变经济发展方式，更加注重节约资源，更加注重环境保护，更加注重改善民生，更加注重社会和谐，全力推动经济社会在又好又快发展基础上实现新的跨越。

主要预期目标是：地区生产总值486亿元，增长20％。地区财政收入51．5亿元，增长21．9％。地方财政一般预算收入27．7亿元，增长20％。全社会固定资产投资460亿元，增长50％。社会消费品零售额153亿元，增长18％。实际利用外资1．2亿美元，增长20％。外贸出口1．4亿美元，增长15％。城镇居民人均可支配收入1万元，增长12％。农民人均纯收入5600元，增长12％。

为实现上述目标，将着力抓好以下9项重点工作：

(一)以沈铁工业走廊为中心，加快推进新型工业化进程

沈铁工业走廊是铁岭经济社会发展的支撑，是实施工业化的载体，是对外开放的窗口，是科技创新的平台，是强市富民的希望，是我市最大最靓丽的名片。把沈铁工业走廊推向优化发展的新阶段，加快实现“两个跨越”，即由工业集中向产业集聚跨越，由发展传统工业向新型工业化跨越。在更高层次上参与国际分工，在更高水平上承接国内产业转移，在更广领域上创造发展商机，把沈铁工业走廊打造成功能最完备、服务最优良、环境最优美、竞争力最强的新型工业化基地。

进一步加强园区建设和管理。完善沈铁工业走廊区域发展规划、功能分区规划和重点园区规划，更加科学地确定发展方向和产业定位。多渠道筹集资金，加快园区基础设施建设，进一步完善园区功能。理顺园区管理体制，加快建立一门代办受理、一次会议审批、一个公章生效“三个一”工作机制，真正把园区建成经济发展的“特区”。

积极培育产业集群。围绕“两大基地”和“五大主导产业”，以重点产品为龙头，以骨干企业为核心，以优势产业为纽带，大力培育特色产业基地。巩固和扩大铁岭县有色金属和装备制造、开原市起重机制造和有色金属、昌图县新型能源和生物化工、调兵山市能源和煤化工、银州区机械加工、清河区食品加工、高新区环保装备和先进制造、开发区橡塑制品和高新技术、西丰县土特产品等产业集聚区。

大力推进企业技术改造和技术创新。继续实施技术改造“1123”，工程：争取省技改贴息资金1亿元，技改投资100亿元以上，重点培育20户骨干企业，实施3 0个重大技改项目。深入开展科技创新年活动，高新技术产值实现107亿元，增长50％。高新区确保完成固定资产投资40亿元，确保50个企业投产。做大做强骨干企业，新增销售收入亿元以上企业30户、10亿元以上企业15户。规模以上工业增加值实现220亿元，增长30％，工业增加值占地区生产总值的比重提升4个百分点。

(二)以项目年活动为载体，加快开放立市步伐

发展必须开放。一个地区发展的空间取决于开放的空间，发展的水平取决于开放的水平，大开放大发展，小开放小发展，不开放难发展。招商引资是扩大开放的主要内容，是摆脱困境的关键举措，是促进发展的重要途径。要坚定不移地实施开放立市战略，加快形成全方位、多层次、宽领域的对外开放新格局。

进一步加大招商引资力度。一是加强项目库建设。立足资源优势、区位优势、环境优势，超前谋划一批市场前景好、带动能力强、产业层次高的大、高、外项目。二是创新招商方式。加快实现由运动式招商向经常性招商转变，由招商引资向招商选资转变。大力推广铁岭县“飞地招商”经验。落实“飞地招商”政策，调动各方面向园区引进项目的积极性，促进资源合理配置。引导相同项目集中摆放，相关产业集中发展，加快实现由分散办企业向集中做产业转变，逐步走上园区共用、项目共引产业共建成果共享的良性发展轨道。三是精心组织好各项重大招商活动，争取引进世界500强企业和国内上市公司来我市投资兴业。四是精心营造有利于投资创业的良好环境，把铁岭建成服务高地、成本洼地、发财宝地。全市确保引进国内外资金350亿元，引进亿元以上项目1OO个。不断扩大出口规模，优化出口产品结构。继续加强对外劳务合作，开拓境外合作新领域。

继续开展项目年活动。坚持一手抓“筑巢引凤”，一手抓企业技术改造；一手抓大项目，一手抓产业集群。重点抓好工业项目，统筹抓好农业产业化、现代服务业、基础设施、社会事业等项目。举全市之力支持大唐集团煤化工项目，争取国家早日核准并开工建设。重点抓好铁岭电厂；期和三期、清河电厂“上大压小”、风力发电、煤矸石发电、金信生物化工、博大生物二期、华扬机械、50万伏输变电设备、掌握客车、中利电缆、金刚水泥、铁新水泥、石油机械、三洋机械、开原雨润、亮中煤田、石油储备等100个重点项目的开工建设和达产达效。对重点项目组成精干的项目推进组，落实责任，跟踪服务，确保项目顺利实施。

(三)以现代农业为方向，加快建设社会主义新农村

加快发展县域经济。县(市)区地区生产总值、财政一般预算收入平均增长3O％以上，规模以上工业增加值平均增长40％以上。加快建设农产品精深加工基地。大力培育龙头企业，固定资产投资2000万元以上产业化项目新增20个以上，重点扶持雨润、赢德、大成、北绿等20个龙头企业，农业产业化总产值达到310亿元，增长20％。继续推进保护地蔬菜、优质水稻、榛子、花生“四个一百万亩”工程，调整优化种植业结构。全面推进农业标准化生产，新增绿色食品标识1O个、无公害农产品标识3O个。新建村外畜牧小区8O个，畜牧业产值实现137亿元，增长17．5％。肉、蛋、奶总产量达到104万吨，增长11．4％。加强动物疫病防控，确保畜牧业生产安全。加强城乡绿化，完成人工造林2O万亩，新增榛林13．8万亩，实现林业产值25亿元，增长47％。大力发展各种形式的农村专业合作经济组织、民间行业协会和民间商会，提高农民的组织化程度，农民专业合作社发展到200家。推广应用100项农业新品种、新技术，提高科技创新对社会主义新农村建设的支撑能力。加强农业基础设施建设，完成农建土石方3000万立方米，建设骨干工程200项，继续争夺省“大禹杯”。进一步提高农业机械化水平，农机总动力达到186万千瓦，综合机械化水平达到5O％。围绕增加农民收入、改善农村民生和保护农村生态环境，着力办好12件实事。1．培训转移农村富余劳动力5万人。2．实施开发式扶贫工程，实现100个村、5万贫困人口脱贫。3．实现农村低保对象4．3万户、8．8万人应保尽保。4．新建农村饮水安全工程100处，解决10万农村人口饮水安全问题。5．完成农村公路建设500公里，新建农村客运站9个。6．落实农村义务教育阶段“两免一补”政策，确保农村学生不因家庭贫困而失学。7．完成大部分村标准化卫生所建设。8．新建和完善乡村文体广场110个。9．新建清洁能源工程10处，完成农村改厕示范工程3000个。10．新建村信息站100个。11．西丰诚信水库建成并投入使用。12．建设乡镇温馨候车站点200个。

(四)以凡河新城区建设为重点，加快推进城镇化

凡河新城区建设是一项前无古人、荫及子孙的伟大事业，是新铁岭大铁岭的标志，也是铁岭的希望和未来。新城区建设的重大现实意义和深远的历史意义，必将随着形势的发展和时间的推移逐渐显现出来。全市上下要倍加珍惜千方百计争取到的有利条件，倍加珍惜千辛万苦创造出的建设成果，不为困难所惧，不为干扰所惑，“咬定青山不放松”，抢抓机遇，乘势而上，把主要精力、工作重心、优势资源尽快转到新城区建设上来，以只争朝夕的精神加快新城区建设步伐。今年完成投资6O亿元，累计投资超过100亿元，建筑工程面积260万平方米，确保实现“三年大变样”的战略目标。

新城区建设重点实施10大工程：1．15平方公里基础设施建设工程。2．水系工程。确保汛期前全线通水，充分展现水城魅力。3．绿化工程。确保一次绿化，一次成形，达到国家园林城市标准。4．莲花湖湿地恢复、凡河湿地自然保护区工程。使新城区成为“建在湿地上的城市”。5．如意湖和天水河景观工程。使如意湖成为镶嵌在钻石状城区上的一颗璀璨明珠，天水河成为流动在城区中轴线上的一道靓丽风景。6．环如意湖建筑群工程。使其成为新城区的标志性建筑。7．商住建设工程。其中“浅水湾一号”达到入住标准。8．公共服务工程。确保技师学院、财税中心、铁岭县高中、九年一贯制学校、市体校9月份竣工并交付使用。9．旅游精品工程。开工建设总投资20亿元的“和谐之都”大型旅游项目。10．物流城建设工程。确保一期工程100万平方米竣工并投入运营。

继续以“五城联创”为载体，进一步加强城市管理，搞好城市经营，增强城市活力，提升城市功能。重点抓好10件实事：1．提高城市绿化水平，争创国家级园林城市。2．新建13个垃圾中转站。3．新建水洗公厕1O座。4．更新改造167处居民住宅二次加压供水设施。5．完成市中水回用一期工程，满足部分企业工业用水。6．打通光荣街。7．继续对小街小巷环境进行综合整治。8．新建铁岭市第六中学。9．推进社区卫生务体系建设，逐步解决看病难看病贵问题。10．按照资金补贴为主、实物安置为辅的原则，解决1万平方米、334户城市廉租房。突出抓好5个副中心城市建设。继续抓好2O个中心集镇建设，完善城镇规划，改善基础设施。

(五)以东北物流城为突破口，加快发展现代服务业

全力支持铁岭东北物流城建设，配合华南城集团精心组织好招商活动。抓好西丰东北土特产品交易中心、开原五金城等招商工作。加快果菜批发市场、开原循环经济物流园、铁岭县物流配送中心等重点物流项目建设。加快建设特色专业市场，继续打造银州区南马路商业一条街和餐饮娱乐一条街。积极发展新型经营业态，规划建设国内领先的大型综合购物中心。以创新体制机制、推进信息化、扶持龙头企业为着力点，深入推进“万村千乡市场工程”，新增村级连锁店100个。以东北物流城建设为重点，尽快把铁岭建成东北物流集散地。以“浅水湾一号”等大型商业住宅建设为重点，大力发展房地产市场。以新城区“和谐之都”大型旅游项目和莲花湖国家级湿地公园建设为重点，做大做强旅游产业。以北方金融后台服务基地建设为重点，努力拓展金融服务业。使现代物流、房地产、旅游、金融服务成为我市现代服务业的四大支柱产业，促进传统产业优化升级，提高现代服务业在国民经济中的比重。落实粮食储备制度，大力推广科技储粮，增强政府对粮食市场的调控能力。加强市场监管和价格监测，维护流通市场秩序，稳定消费价格，严厉打击制假售假行为，保证消费安全。

(六)以深化改革为动力，加快实现体制机制创新

进一步深化国企改革。做好精工、开原纸业等重点企业的改制以及铁阀集团计划内破产和壳企业依法破产工作，加大水泥纸袋、橡胶、清河建材等破产企业招商引资力度。做好长城、信达公司债权回购的资金催缴和资产变现工作，盘活国有闲置资产。建立国有资本经营预算制度，强化国有资产监管。深化投融资体制改革。优化金融环境，加强诚信建设，充分发挥国有商业银行融资主渠道作用。继续搞好市商业银行改组改制，实行增资扩股，建立资本补充机制。推进农村金融体制改革和创新，搞活农村金融。强化资本运作，完善融资平台，在直接融资上下功夫，推动具备条件的企业上市，争取发行企业债券和短期融资债券，不断拓宽新的融资渠道。进一步加强担保体系建设，提高中小企业融资能力。积极稳妥地搞好农村综合改革和农垦企业改革，增强发展活力。认真落实“三项补贴”等惠农政策，保护和调动农民的生产积极性。稳定和完善土地承包关系，在尊重农民意愿的基础上，推进土地流转和股份合作经营。巩固集体林权改革成果，加快推进林业产业化步伐。加大国有土地招拍挂力度，优化国土开发格局。积极稳妥地搞好事业单位机构改革，扩大事业单位的用人自主权和分配自主权。适应市县两级行政中心南移的新形势，进一步明确市、县、区的职责，理顺中心城区的管理体制。

(七)以节能减排为重点，加快转变经济发展方式

节能减排是节约能源资源、保护生态环境的重要途径，是转变经济发展方式的必由之路，是促进科学发展的重要抓手。坚持开发与节约并举、节约优先的方针，以提高资源利用率为核心，以节能、节水、节电、节地为重点，把节约能源资源贯穿于生产、流通、消费的各个环节和经济社会的各个领域，尽快形成政府为主导、企业为主体、全社会共同推进的节能减排工作格局。层层建立节能减排工作责任制和指标考核体系，将其作为衡量经济发展成效和政绩的一条重要标准。正确处理发展与环境的关系，拒绝落后生产力向我市转移。对2O户严重环境违法企业予以关停取缔，对15户环境违法企业限期治理，对87户环境违规企业实行挂牌督办。大力加强辽河、凡河、柴河、清河等重点流域生态保护。加快推进集中供热，确保今冬铁岭电厂向凡河新城区供暖。切实加强环境监管，严格执行“三同时”制度，建设绿色工业园区。推广应用节能减排的先进适用技术，建设好环保产业园，积极发展循环经济，努力建设资源节约型、环境友好型社会。万元生产总值能耗下降5％，水耗下降4％，二氧化硫、化学需氧量以2005年为基数分别削减7．2％和9．7％，争创全国环保模范城。

(八)以改善民生为重点，加快构建和谐社会

坚持以人为本，下力气解决人民群众最关心、最直接、最现实的利益问题。实施全民创业促就业工程。实名制培训下岗失业人员和进城务工农民2万人，实现再就业、5万人，确保零就业家庭至少有一人稳定就业，城镇登记失业率控制在5％以内。建立健全大学毕业生就业帮扶体系，努力拓宽就业渠道。不断完善社会保障体系。征收“五险合一”社保资金16．1亿元，实现企业养老保险市级统筹，探索建立农村养老保险制度，确保老有所养。加快构建以基本医疗保障为主体，以大病统筹为重点，兼顾多层次医疗需求的城镇居民医疗保障体系，确保病有所医。逐步扩大失业保险、工伤保险、生育保险覆盖面，确保各项社会保险金及时足额发放。建立企业职工工资正常增长机制和农民工工资支付保障机制，逐步提高扶贫标准和最低工资标准，千方百计增加城乡居民收入。完善城乡最低生活保障制度，努力做到应保尽保，并积极创造条件提高保障标准。对城市低保边缘户实施救助，继续改造农村低保户危房，逐步形成基础性救助和专项救助相结合、政府救助和社会救助相结合、城乡一体化的综合性救助体系。

全面发展各项社会事业。优先发展教育，逐步实现教育均衡发展，促进教育公平。继续开展“教育年”活动，确保以县为主体的九年义务教育和“两类新三片”普九规划达标，做到学有所教。合理配置优势资源，做大做强高中教育。大力发展职业教育鼓励和规范社会办学，争创1所国家级高职示范院校和1所国家级重点中等职业学校，打造2—3个省级职教品牌专业，努力将我市建成有影响的职业教育基地。加快发展高等教育，积极创建市属本科大学。加大投入力度，着力改善学校基础设施和办学条件，新建农村九年一贯制学校10所，创建省级标准化九年一贯制学校5所，维修改造校舍5万平方米。继续开展捐资助教活动，募集教育基金1000万元。深化教育改革，加强教师队伍建设，逐步提高农村学校师范类本科毕业教师比重，全面提高教育教学质量。进一步健全公共卫生服务体系，提高重大疫病防控及突发性公共卫生事件应对和处置能力。不断完善新型农村合作医疗制度，全面完成乡镇卫生院建设，深化卫生院管理体制改革，不断改善农村卫生条件。坚持政府主导、社会参与、市场化运作的原则，加快完善城市社区卫生服务体系。积极发展中医药事业。加强医护队伍建设，提高医疗服务质量。加强乡镇文化站和基层文化基地建设，完成224个广播电视村村通工程，实现中心城区有线广播电视数字化整体平移。挖掘和保护铁岭特色文化，打造文化品牌，提升文化品位，继续推进文化名城建设。搞好银冈书院国家级文物保护单位申报和第三次文物普查工作，完成铁岭图书馆建设并投入使用。研究制定文化产业发展规划和扶持政策，壮大文化产业。广泛开展以迎奥运为主题的全民健身活动，继续实施“百万农民健身工程”。继续稳定低生育水平，提高出生人口素质。大力加强社会公德、职业道德、家庭美德和个人品德建设，培育市民意识，提高公民素质。深入开展各种形式的群众性精神文明创建活动，形成良好的社会风尚，争创全国文明城市。

加强社会管理，维护社会稳定。全面贯彻党的民族政策，扶持少数民族乡镇加快经济社会发展。坚持宗教工作的基本方针，发挥宗教界人士和信教群众在促进经济社会发展中的积极作用。进一步落实信访投诉工作责任制和责任追究制度，深入开展社会矛盾纠纷排查调处工作。启动“安全生产企业标准化创建年”活动，进一步落实企业安全生产主体责任。加大安全生产监管和事故查处力度，切实解决守法成本高、违法成本低问题。完善应急预案体系，建立健全突发事件监测、预警和信息报告制度，加强应急救援队伍建设，提高处置各类突发事件的能力。全面落实社会治安综合治理各项措施，继续加强“三基”工程建设，深入开展阳光警务工程，严厉打击各类违法犯罪活动，加快平安建设，构建和谐铁岭。

(九)以提高行政效能为目标，加快转变政府职能

牢固树立科学发展观和正确的政绩观，完善政府系统绩效考评体系，为提高行政效能提供制度保证。建立健全行政问责制度和责任追究制度，切实提高政府公信力和执行力。强化行政效能监察，认真受理行政效能投诉，对行政效能低下、人民群众意见比较集中的部门实行质询制度，主要领导要向群众公开道歉并追究有关人员的责任。加大政务督查工作力度，继续开展“选优评差”活动，奖勤罚懒，促进落实。巩固第五轮行政审批制度改革成果。加强市县乡三级公共行政服务系统建设，逐步实现审批和收费事项全部进入公共行政服务中心集中办理，杜绝体外循环和暗箱操作。坚持和完善“限时办结、超时默认”、“一门受理、一门送达”、审批与管理分设等制度，努力提高办事效率。全面推进政务公开，加强电子政务建设，健全政府网站体系，完善网站服务功能，方便群众办事，降低行政成本，努力建设服务型政府。

自觉接受人大及其常委会的依法监督和人民政协的民主监督，认真执行人大及其常委会的各项决议、决定。加强和改进人大代表建议、政协提案办理工作，提高办理质量和代表、委员的满意度。加强与民主党派、工商联、无党派人士、人民团体及社会各界人士的联系，广泛听取他们的意见和建议。深入贯彻依法治国方略，加快建设法治政府。强化依法行政教育，提高依法行政意识和能力。加强对规范性文件的监督管理，从制度上规范行政权力。落实行政执法责任制，完善行政执法程序，强化政府法制监督和行政复议工作，规范行政执法行为。公正办理仲裁案件，维护社会公平正义。建立和完善重要行政决策听证制度、合法性论证制度、集体决定制度、实施情况反馈制度和决策责任追究制度，努力提高科学决策水平。坚持标本兼治、综合治理、惩防并举、注重预防的反腐倡廉方针，继续抓好广大公务员的廉洁自律，坚决纠正各种不正之风，严肃查处违法违纪案件。切实加强作风建设，以求真务实、严谨高效的作风推动各项工作的落实。

各位代表，当前铁岭的发展已经站在了一个新的历史起点上，正处于经济社会发展全面转入科学发展轨道的关键时期。新的征程已经开始，风劲潮涌，正当扬帆破浪；任重道远，更需快马加鞭。让我们高举中国特色社会主义伟大旗帜，更加紧密地团结在以胡锦涛同志为总书记的党中央周围，在中共铁岭市委的坚强领导下，众志成城，埋头苦干，为实现经济社会跨越式发展，为夺取新铁岭大铁岭建设的新胜利而努力奋斗！
